# Electrons in one dimension

**DOI:** 10.1098/rsta.2009.0226

**Published:** 2010-03-13

**Authors:** K.-F. Berggren, M. Pepper

**Affiliations:** 1 Theory and Modelling, Department of Physics, Chemistry and Biology (IFM), Linköping University, SE-58183 Linköping, Sweden; 2 London Centre for Nanotechnology and Department of Electronic and Electrical Engineering, University College London, Torrington Place, London WC1E 6BT, UK

**Keywords:** nanotechnology, lattices, materials

## Abstract

In this article, we present a summary of the current status of the study of the transport of electrons confined to one dimension in very low disorder GaAs–AlGaAs heterostructures. By means of suitably located gates and application of a voltage to ‘electrostatically squeeze’ the electronic wave functions, it is possible to produce a controllable size quantization and a transition from two-dimensional transport. If the length of the electron channel is sufficiently short, then transport is ballistic and the quantized subbands each have a conductance equal to the fundamental quantum value 2*e*^2^/*h*, where the factor of 2 arises from the spin degeneracy. This mode of conduction is discussed, and it is shown that a number of many-body effects can be observed. These effects are discussed as in the spin-incoherent regime, which is entered when the separation of the electrons is increased and the exchange energy is less than *kT*. Finally, results are presented in the regime where the confinement potential is decreased and the electron configuration relaxes to minimize the electron–electron repulsion to move towards a two-dimensional array. It is shown that the ground state is no longer a line determined by the size quantization alone, but becomes two distinct rows arising from minimization of the electrostatic energy and is the precursor of a two-dimensional Wigner lattice.

## 1. Introduction

**Figure 1.**
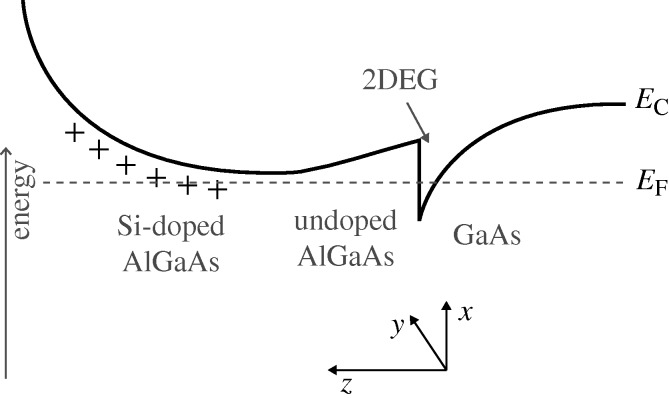
Schematic energy diagram for the conduction band (*E*_C_) in a modulation-doped GaAs/GaAlAs heterostructure. Because of the different bandgaps in the two materials, there is a bandgap offset at the interface. Electrons from the doped region are transferred to the interface where they are trapped because of electrostatics in a narrow effectively triangular potential in the *z*-direction; the motion of electrons along the (*x*,*y*)-interface remains free. The motion in the *z*-direction is quantized into discrete states *ϕ*_*n*_(*z*). When only the lowest state *n*=0 is occupied, as in the cases discussed here, the electrons thus occupy the states 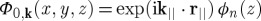, where ***k***_||_⋅**r**_||_=*k*_*x*_*r*_*x*_ + *k*_*y*_*r*_*y*_. In all states with energies below the Fermi energy *E*_F_ being occupied, the system therefore forms a (quasi-) two-dimensional electron gas (2DEG) whose density may be varied by applying a gate voltage *V*_g_ (left-hand side of the figure).

Because of the development of different growth-techniques technology in the early seventies, such as metal organic chemical vapour deposition and, in particular, molecular beam epitaxy, semiconductor materials and structures with reduced dimensionality and extreme smallness may be grown with high precision (for some early overviews see Dingle (1987) and Weisbuch & Vinter (1991)). Important examples are composite materials consisting of alternating layers or lateral sheets of different semiconductor materials that form a superlattice, i.e. the system is periodic in one direction and continuous in the other two. As the bandgaps of the constituents are different, there is a periodic array of potential wells. If there is only one well embedded between two other semiconductor layers of wider bandgap, and this well is deep and narrow enough, carriers may be trapped in discrete quantum states, i.e. we have effectively a quasi-two-dimensional quantum well (QW) of rectangular shape, for example, a GaAs QW embedded in AlGaAs. The motion across the well is quantized, while it is free in the lateral directions. The thickness of such a QW is typically approximately 5–20 nm. There are also alternative ways of achieving confinement. By means of modulation doping, a single triangular potential well trapping a two-dimensional electron or hole gas buried at the interface of, for example, GaAs and GaAlAs, may be created, as shown in figure 1 for the case of electrons (Weisbuch & Vinter 1991). Effectively two-dimensional systems may also be achieved in Si metal–oxide–semiconductor field-effect transistors (MOSFETs; for a comprehensive review, see Ando *et al.* 1982). In the present context, we also mention the dimensional crossover that may be defined in special GaAS field-effect transistor (FET) devices by means of Schottky gates and electrostatic confinement (Poole *et al.* 1982). QW-based high-mobility semiconductor heterostructures are now commonplace in science and technology. For example, they are the basis of laser diodes found in compact disc players, sensitive microwave receivers, high-speed and opto-electronics, etc.

Having achieved the two-dimensional systems in the 1970s, a further reduction of dimensions was achieved in the 1980s by employing different micro-fabrication techniques such as etching, electron and/or X-ray lithography, and doping/ion implantation have allowed a further reduction of the dimensionality. The two-dimensional structures may be turned into a stripe by removing material by etching, such that the motion of carriers becomes restricted in one more direction, let us say the *y*-direction; while they may still travel freely along the stripe, i.e. the *x*-direction (e.g. Kirk & Reed 1989, 1992; Beenakker & van Houten 1991; Davies 1998). Effectively the systems become quasi-one-dimensional.

**Figure 2.**
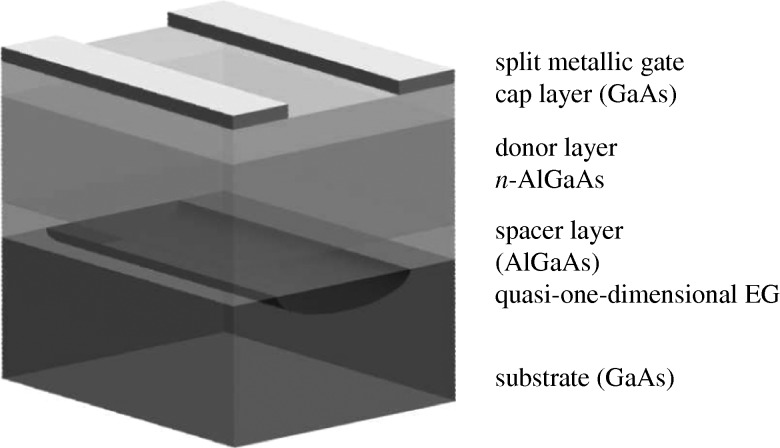
Schematic of a quantum wire embedded at the interface of a gated modulation-doped GaAs/A*l*_*x*_Ga_1−*x*_As heterostructure (typically *x*=0.33). Top layer regions define the split metallic gate that acts to confine electrons electrostatically to the ungated regions thus forming a (quasi-) one-dimensional electron gas (EG). To operate the device, two voltages *V*_g_ and *V*_sd_ are applied separately to the gated regions; *V*_g_ regulates the effective width and electron density of the electron wire, while *V*_sd_ is the bias potential (the potential drop) between the source and the drain. A finite *V*_sd_ induces a current to flow through the wire (quantum point contact). Typically, the electron gas is formed at about 100 nm below the surface and has a mobility of approximately 4×10^6^ cm^2^ V s^−1^. The lithograpic width is normally in the range of approximately 0.3–1 μm and the length is approximately 0.3–1.5 μm.

**Figure 3.**
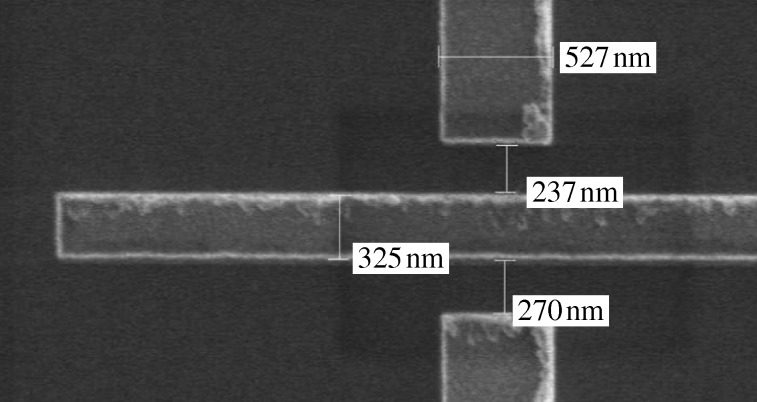
Scanning electron micrograph (SEM) picture of an enlarged section of a patterned GaAs/Al_*x*_Ga_1−*x*_As device with two quantum wires (QPCs) in parallel. The three bars are metallic gates used to control the effective width and subband occupations by applying different gate voltages. The source–drain voltage is applied to the the left and right ungated sections.

While etching, ion implantation, etc. may introduce various imperfections, the first working alternative one-dimensional lateral confinement scheme was introduced by Thornton *et al.* (1986) using split Schottky gates. On applying a negative voltage *V*_g_ to a split top gate, electrons under the gated regions are depleted, leaving a narrow stripe (wire) undepleted, as shown schematically in figure 2. The role of imperfections are generally reduced in this way. Another important feature of this confinement technique is that the width of the stripe and electron density may be varied continuously by varying *V*_g_. Systems fabricated in this way are also very versatile. Wires may be connected to electron reservoirs that serve as source and drain, as shown in figure 3 for a particular device with two parallel wires.

By applying a voltage difference *V*_sd_ between the reservoirs, a current through the wires may be induced. On a relative scale, the voltage drop may be made large, which allows novel studies of quantum transport phenomena in the nonlinear, non-ohmic regime in combination with applied parallel/perpendicular magnetic fields. In summary, the flexibility of split-gate high-mobility semiconductor devices continuously generates a number of fundamental issues in low-dimensional physics and, consequently, there is a rich literature that is too large to be covered here. In the following sections, we will outline some key issues related to quantization, transport and the so-called 0.7 conduction anomaly. For a recent survey of measurements and different interpretations of this phenomenon, see Pepper & Bird (2008).

There is a further advantage of, for example, split-gate GaAs/AlGaAs wires (also known as quantum point contacts; QPCs) when it comes to theoretical modelling. A fundamental property of systems of this kind is that, on the scale of the typical interelctronic separations, the crystal lattice can be treated as a smooth background, whose role is to set the effective mass m* of the electrons and to reduce the effective Coulomb interactions by the static dielectric constant of the semiconductor. Therefore, the effective mass approximation (EMA) applies. Another positive aspect is the high mobility that may be achieved in real devices. Hence, ballistic transport theory is many times more than adequate. All of this evidently makes modelling less cumbersome. The Kohn–Sham local spin-density approximation (LSDA; Parr & Yang 1989), which is so successful when applied to semiconductor superlattices, tunnelling devices, high-mobility electron transistors (HEMTs) etc., should also therefore be a good starting point in the present context. In LSDA, one assumes that a system behaves locally as an electron gas, an assumption that fits very well into our story.

## 2. Nature of single-electron states and conductance

**Figure 4.**
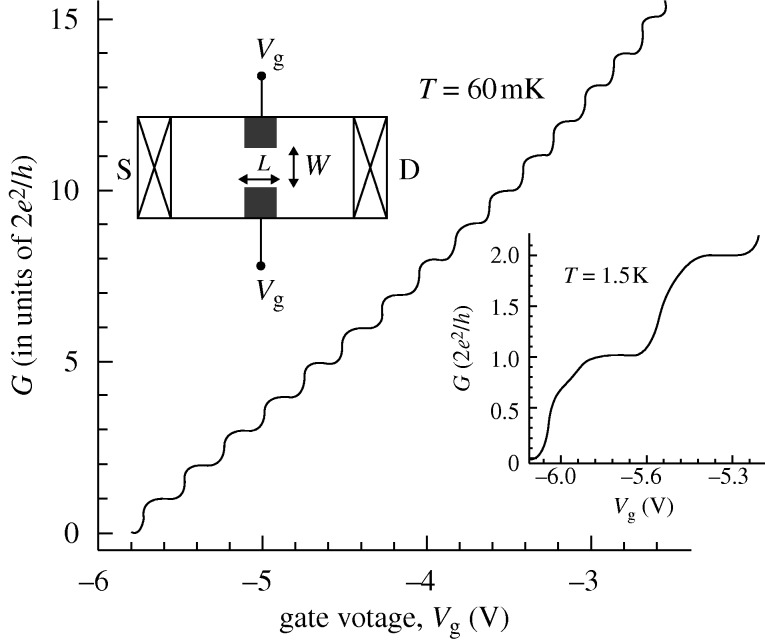
Measured conductance *G* at *T*=60 mK as a function of gate voltage for a QPC fabricated from high-mobility GaAs/Al_*x*_Ga_1−*x*_As. The upper inset is a schematic view of the split-gate device used in the measurements; S and D represent the source and drain contacts. The lithographic length (*L*) of the split gate is 0.4 μm and the width (*W*) typically 0.75–0.95 μm. The lower inset shows the first and second conduction plateaux at *T*=1.5 K (adapted from Thomas *et al.* 1998; courtesy K. J. Thomas, Cavendish Laboratory, UK).

When sufficiently narrow, wires display strong quantum characteristics at low temperatures because of transverse quantization and the formation of subbands with threshold energies *E*_*n*_. Thus, magnetic depopulation of one-dimensional subbands were observed in the first generation of split-gate devices (Berggren *et al.* 1986). Subsequently, quantized conduction was discovered in 1988 in improved short quantum wires (QPCs), i.e. when sweeping the gate voltage *V*_g_, conductance steps of 2*e*^2^/*h* were observed (van Wees *et al.* 1988; Wharam *et al.* 1988). The experimental data in figure 4 show a large number of well-resolved conductance steps.

The gross features of the conductance steps may be understood within a simple model for non-interacting electrons. This may seem surprising, as it has often been argued that standard Fermi-liquid theory would not apply to low-dimensional systems as here. As it turns out, however, the simple model provides a good starting point. If we assume that the wire is extended, the one-electron states for the lateral motions are 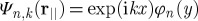, where the plane wave refers to the translational motion along the wire and *φ*_*n*_(*y*) to the *n*th transverse sublevel state. In the EMA *φ*_*n*_(*y*) then obeys the Schrödinger equation2.1
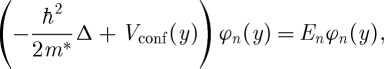

where *E*_*n*_ is the sublevel energy and m* is the effective mass of the GaAs conduction band. The term *V*_conf_(*y*) is the transverse confinement potential, usually of parabolic form at low fillings (cf. 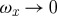 in the saddle potential below). The energy of an electron occupying the *n*th subband is therefore 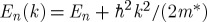. The subbands are all occupied up to a common Fermi energy *E*_F_ and the total wave function for the system of non-interacting electrons is a single Slater determinant.

Let us first look at a single subband. If we integrate over the (normalized) transverse mode, the velocity *v*_*x*_ for a particular subband state is 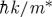. Assume now that there is a net flow of electrons through the wire because there are more electrons flowing in one direction than the other. Integrating *ev*_*x*_ over the *k*-states within a small energy window Δ*E* at *E*_F_, we obtain the corresponding current *I*=(*e*/*h*)Δ*E*. Now let Δ*E* be related to the source–drain bias as Δ*E*=*eV*_sd_, which gives *I*=(*e*^2^/*h*)*V*_sd_. The conductance for an occupied subband is therefore *G*=(*e*^2^/*h*) per spin direction. Including all the occupied subbands and the spin directions, we finally have the total conductance *G*=*N*(2*e*^2^/*h*), where *N* is the number of occupied subbands. Every time a subband becomes occupied as *V*_g_ is swept as in figure 4, there is thus a conductance step of (2*e*^2^/*h*). For a more detailed derivation of *G*, together with definitions of the AC and DC conductances *G*_AC_=∂*I*(*V*_sd_)/∂*V*_sd_ and *G*_DC_=*I*(*V*_sd_)/*V*_sd_, we refer to appendix A.

**Figure 5.**
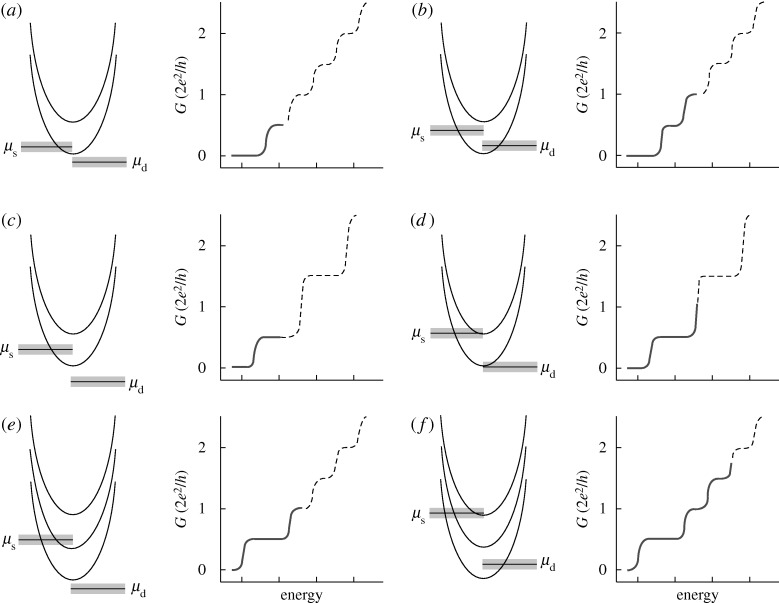
Spin-degenerate subbands 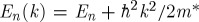 in a parabolic quantum wire with subband spacings 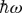 and the effect of source–drain bias on the AC conductance *G* for spin-degenerate electrons. Thermal broadening of the order approximately 4*k*_B_*T* is indicated by the marked area surrounding *μ*_s_ and *μ*_d_. (*a*,*b*) The source–drain bias is half the subband spacing, giving differential conductance steps in integer multiples of (*e*^2^/*h*)/2. (*c*,*d*) The source–drain bias equals the subband spacing, which results in conductance steps only at *G* = (*n*+1/2)2*e*^2^/*h*, with *n*=1,2,3,…. (*e*,*f*) *eV*_sd_ is 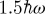 where integer plateaux occur. The corresonding conductances (solid traces) are shown on the right-hand side next to the subbands. In the case of spin splitting due to a magnetic field and/or spontaneous spin polarization, the conductance plateaux are split according to equation (A6). Quarter plateaux, a signature of spin polarization, may then be observed (courtesy T.-M. Chen, Cavendish Laboratory, UK).

The elementary electron-gas model evidently accounts quite well for the presence of 2*e*^2^/*h* plateaux in figure 4. It would also predict that the conductance plateaux split into half-plateaux due to Zeeman splitting and magnetic depopulation when a magnetic field is applied, as first observed by Wharam *et al.* (1988). The model may also be used to predict the conductance at large bias. New types of half-plateaux occur as the occupancy of left- and right-travelling states are varied via *V*_sd_, as shown in figure 5 for spin-degenerate electrons (see appendix A). Furthermore, if the levels are Zeeman split by an applied magnetic field, additional splittings into quarter-levels occur at large bias, as observed recently (Chen *et al.* 2008). This may also happen already at zero magnetic field if there is a spontaneous spin polarization triggered by electron interactions, a possibility that we will return to.

The quasi-one-dimensional electron model above applies to long wires. The transmission for the different states is assumed to be either zero or one. For shorter wires, i.e. QPCs, the transmission increases gradually from zero to one with increasing energy. The reason is that the features of QPC potential are quite smooth. A commonly used model is the saddle-point potential2.2
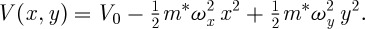



Here, *ω*_*x*_ and *ω*_*y*_ are related to the curvatures of the potentials in the *x*- and *y*-directions and *V*_0_ is the potential at the saddle maximum. The parabolic saddle potential accounts well for the normal integer quantized conductance in a QPC on a one-electron level. It also accounts for the gross features of the soft intermediate regions because it allows for substantial tunnelling for soft potentials (Büttiker 1990). Typical values for 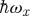 and 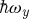 are in the range of approximately 1–3 meV, as obtained from bias spectroscopy (Martín-Moreno *et al.* 1992). For wide constrictions, *ω*_*y*_/*ω*_*x*_ approximately 1–2, the conductance steps rise gradually. The local density of states (LDOS) within the QPC is then broadened vis-à-vis the ideal one-dimensional form with its characteristic 
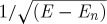
 singularities associated with each subband (Jaksch *et al.* 2005). For higher ratios, the QPCs become effectively more narrow and the transmission coefficients more like step functions as for the infinite wire discussed above. At the same time, the LDOS approaches the ideal one-dimensional form. In summary, the saddle potential model for QPCs works well in predicting conduction plateaux and, in most cases, the shape of intermediate regions. An important deviation is, however, noticeable below the lowest plateau in figure 4 as well as a more faint one below the second plateau. These features may not be captured by independent electron modelling, as will be discussed in the following section.

## 3. The 0.7 conduction anomaly

**Figure 6.**
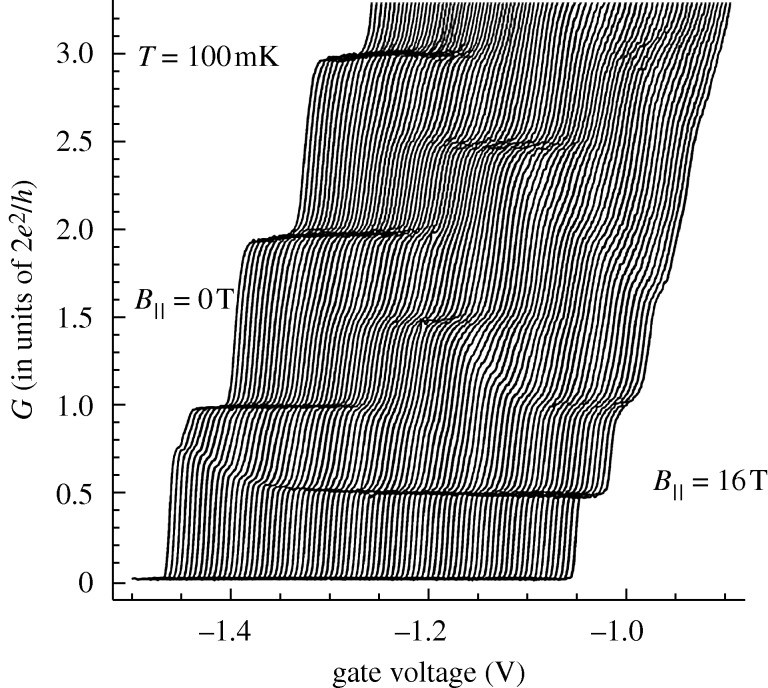
Conductance of a one-dimensional channel is plotted against split-gate voltage as the magnetic field, which lifts the spin degeneracy, is increased from 0 to 16 T; the temperature of the measurement is 100 mK. The individual plots are moved along the voltage axis for clarity. The drop in the 0.7 to 0.5 is clear, as is the creation of the spin-split levels as the field increases.

As discussed earlier, the basic one-electron theory of conduction describes the quantization in units of 2*e*^2^/*h* going to *e*^2^/*h* when the spin degeneracy is lifted by the magnetic field. It was therefore quite surprising when Thomas *et al.* (1996) showed that a feature below 2*e*^2^/*h*, which they had consistently observed, had a distinct physical origin rather than arising from scattering events. Earlier work on lower quality heterostructures showed random structure due to scattering and associated interference in the channel. As this feature, which could vary in appearance from a resonance to a plateau, was near the value 0.7(2*e*^2^/*h*), it was termed the 0.7 structure or feature (Pepper & Bird 2008). The initial investigation showed that, as the magnetic field in the plane was increased, the 0.7 smoothly decreased in value to saturate out at the normal spin-split value of *e*^2^/*h*, i.e. 0.5(2*e*^2^/*h*), and the smoothness of the drop strongly indicated that the origin of the structure arose from the spin of the electrons, see figure 6.

Other aspects of the one-dimensional system were investigated in this work, and it was shown that the Lande *g* value increased as the carrier concentration decreased; such enhancement usually arises from the electron–electron interaction that then seemed responsible for both the *g* value enhancement and the 0.7 feature. As the temperature decreased below 5 K, the 0.7 appeared, as did the normal subband quantization, but then disappeared below 1 K by appearing to merge with the 2*e*^2^/*h* plateau. The temperature dependence could be fitted to an Arrhenius plot, which indicated that the 0.7 state would be excited out of the spin-degenerate 2*e*^2^/*h* (Kristensen *et al.* 2000). Such experiments indicated that the 0.7 arose from a tendency to spin polarization, which was completed by application of a magnetic field. A bias voltage and temperature play a similar role.

Among other experimental investigations was the behaviour of the zero bias anomaly in the differential of the current versus the source–drain voltage *V*_sd_, where a maximum is found at zero *V*_sd_, which increases as the temperature drops and the 0.7 rises and disappears. This was consistent with a possible Kondo effect that, it was suggested, arose from a trapped electron in the channel caused by a weak reflection at the exit region (Cronenwett *et al.* 2002; Hirose *et al.* 2003; Meir 2008). This possibility was investigated by Sfigakis *et al.* (2008), who fabricated a device in which the split gates had curved ends, and found that by applying a suitable offset bias as a voltage, the device could be switched from a one-dimensional channel to a quantum dot. It was found that, when the device was between a quantum dot and a one-dimensional channel, a strong feature was found at *e*^2^/*h*, exactly where a discrete level corresponding to a spin-polarized state would give conductance structure. The resonant shape of the feature was also as expected, as was its characteristic Kondo-like rise as the temperature dropped. Application of a magnetic field split the zero bias anomaly peak, as seen in other systems, and the source–drain voltage separation of Kondo peaks was determined by the Zeeman splitting of the spin levels. However, further work on the zero bias anomaly showed that, if there were a splitting of the spin levels in the channel due to the repulsion of the two spin directions, then the temperature dependence of this effect could also explain the zero bias anomaly. The Kondo explanation for the 0.7 relies on it being what should be a 0.5, but displaced by a small current in parallel with the Kondo current. However, the work of Sfigakis *et al.* showed that the Kondo effect does give the expected behaviour at 0.5, and that this Kondo effect is additive to the normal 0.7 behaviour, that is, the two effects are independent and do not interfere in any way. If the 0.7 is attributed to a trapped electron, then the existence of an extra trapped electron should modify the observed behaviour, which is not observed. Evidence has been presented for a bound state just when the channel is first formed, but this corresponds to a much smaller value of conductance than 0.7 (Yoon *et al.* 2009). The inferred remnant spin splitting at zero magnetic field was found inconsistent with a Kondo model, however, and appears in agreement with models that predict a static polarization in the QPC (Starikov *et al.* 2003; Berggren & Yakimenko 2008).

A number of theoretical papers have been published suggesting that a spin polarization does occur despite the theorem of Lieb and Mattis that the ground state of a one-dimensional system must be non-magnetic (see appendix B). However, it is not clear if this theorem holds in the presence of an applied voltage or for a nanostructure that is not one-dimensional in a strict mathematical sense (Jaksch *et al.* 2005). By following the evolution of the conductance plateaux with the source–drain voltage and magnetic field, it has been observed that, in the region of the 0.7, the two spin levels are spontaneously split, and the lowest spin level is occupied, whereas the upper spin level appears pinned close to the source potential. This upper spin level is then non-degenerate. The conductance is now determined by the full transmission of one spin level giving 0.5(2*e*^2^/*h*) and partial transmission of the other with a smaller contribution to the overall conductance. This situation corresponds to a greatly enhanced *g* value that increases as the population of the electrons in the lowest spin level increases, and the upper spin level remains at the source potential. Eventually, as the carrier concentration increases, the upper level is pulled below the source potential and the normal 2*e*^2^/*h* plateau is obtained. As the temperature decreases, so the upper spin level moves down in energy to become close to, and eventually at, the source potential, so becoming degenerate, resulting in the increase and eventual disappearance of the 0.7 into the 2*e*^2^/*h* plateau at the lowest temperature (Graham *et al.* 2007).

**Figure 7.**
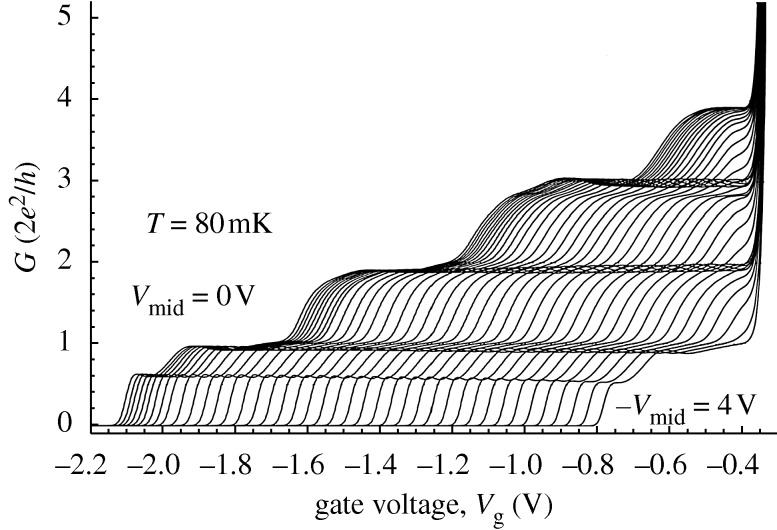
The conductance of a device is plotted against split-gate voltage with the carrier concentration lowered by a voltage applied to a gate over the middle of the channel, which is termed *V*_mid_. As the carrier concentration is decreased (left to right), the 0.7 drops towards 0.5. The temperature of the measurements is 80 mK.

The effect of varying the carrier concentration has been investigated, and it was found that, as this was reduced, so the 0.7 moved down towards the fully spin-polarized value of 0.5 (Thomas *et al.* 2000), as in figure 7.

Confirmation of the fully spin-polarized state was provided by the absence of any change in this plateau when a magnetic field was applied. The disappearance of the 0.7 at the lowest temperatures indicates that it is not a ground state but is rather an excited state. This seems to be a more general property of spin levels as, if spin polarization is produced by a magnetic field, then, as the temperature is increased, so the 0.5 increases towards 0.7. This result opens up the possibility that the 0.7 is a fundamental property of one-dimensional nanostructures when degenerate spin levels are present, and arises from spin repulsion, giving a tendency to ferromagnetic alignment when being excited.

Thermal measurements have also been performed in the region of the 0.7. Chiatti *et al.* (2006) measured the thermal conductance of the electrons and found that the Wiedmann–Franz ratio breaks down, whereas it is found in all other values of conductance. These authors showed that the thermal conductance takes the characteristic of a conductance of *e*^2^/*h* and not 0.7, implying that, on a model of spin-split subbands, the number of carriers in the upper spin subband, which is located just above the source, increases as the temperature falls and the band moves closer to the source. The net effect is that the thermal energy carried by the electrons is substantially independent of the temperature, as the diffusion current from the cold to hot ends is balanced by a thermal current the other way. The result could be a reduction or absence of thermal conductance due to this subband. The thermopower has been measured in the regions of the 0.7 and the integer conductance plateaux (Appleyard *et al.* 2000). Application of the Mott formula indicates that the thermopower *S* should be zero when the conductance is not changing as the Fermi energy changes, i.e. the plateaux. Measurements on a sample with a well-defined 0.7 plateau showed that, as expected, *S* was zero when the conductance took quantized values; however, in the region of the 0.7, it was high and not constant, but when a magnetic field was applied and the 0.7 dropped towards 0.5, so *S* dropped towards zero, as implicit in the Mott formula. At the time, this was an inexplicable result; however, more recently, the importance of spin entropy in thermoelectric power measurements has been realized. Essentially, if spin polarization takes place in the channel, then, as electrons exit the channel, they revert to a spin-degenerate state and their electrochemical potential will decrease, so liberating thermal energy. The result of this process is to increase the temperature of the cold end of the sample above what it would be in the absence of the spin polarization; this reduction in the temperature difference produces an enhanced thermopower. Application of the magnetic field maintains the spin polarization as electrons exit the channel and agreement with the Mott formula is found.

Noise measurements have been performed in the region of the 0.7 and have shown that there are two channels, one of which is transmitted much more substantially than the other. This result is as expected for a spin polarization, but, at present, magnetic field measurements have not been performed, so this conclusion cannot be completely confirmed (Roche *et al.* 2004).

Conductance measurements have been performed on holes in high-quality GaAs heterostructures, and the 0.7 was found to exist in this system. The dependence of this on spin was displayed by the spontaneous spin splitting varying as the *g* value, which is anisotropic in the surface plane (Danneau *et al.* 2008). Other semiconductors have also revealed its presence, but not as clearly as the GaAs system, which has very high quality layers.

It is clear from the above discussion that the 0.7 is a fundamental property of one-dimensional systems and that a clear understanding of its origin has not fully emerged.

## 4. Spin effects in one-dimensional channels

A number of investigations have been carried out in spin effects in general. Although interaction effects are strongest at the lowest values of carrier concentration, and consequently in the first subband, there is considerable evidence of their existence in the higher energy subbands. For example, the initial investigations of the 0.7 anomaly also provided evidence of the zero field spin splitting, giving rise to structure at 1.7(2*e*^2^/*h*), although this is not as pronounced as the 0.7 that is often present in conductance data. These features are captured by self-consistent spin-polarized LSDA calculations (Starikov *et al.* 2003).

As stated previously, in the presence of a magnetic field, the quantized plateaux separation of 2*e*^2^/*h* is reduced to *e*^2^/*h*. In addition, a reduction in the value of d*I*/d*V*_sd_, which in the ohmic regime takes the normal quantized plateau values, can be achieved by increasing the source–drain voltage *V*_sd_. This results in splitting of the plateaux into two, which are separated by *e*^2^/*h*, and in the presence of a magnetic field, which lifts the spin degeneracy, this becomes *e*^2^/2*h*, i.e. 0.25(2*e*^2^/*h*)—see also appendix A. However, the principal value of lifting the momentum degeneracy is that the voltage drop across the channel is *V*_sd_ and the discrete level splits into two, which are separated by *V*_sd_. The change in conductance with the splitting can be measured, and hence it allows *V*_sd_ to be correlated with the same change in potential that the split-gate voltage produces. In the absence of such a technique, the change in energy of the various one-dimensional levels cannot be ascertained. This technique has enabled the increase in the Lande *g* value to be measured as the carrier concentration decreases.

**Figure 8.**
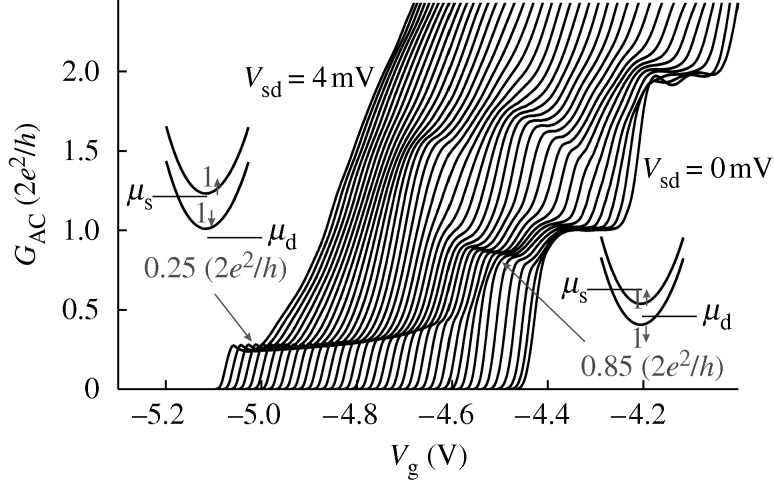
The differential conductance *G*_AC_ is shown against split-gate voltage as the source–drain voltage *V*_sd_ is increased. As seen, the structure at 0.25 remains after the other plateaux have been removed by the source–drain voltage. The insets on either side of the figure show the location of spin-split bands with reference to the source–drain potentials for both 0.25 and 0.85 structures.

As *V*_sd_ increases, the 0.7 feature moves up between 0.75 and 0.85, (always in units of 2*e*^2^/*h*), the exact value depending on the system (figure 8).

**Figure 9.**
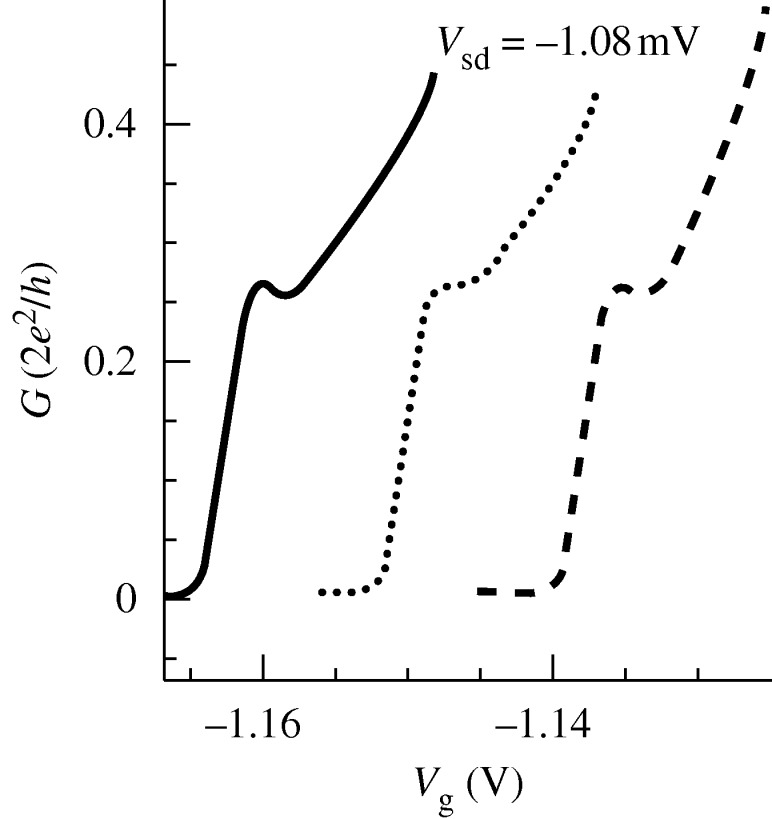
The differential conductance as in figure 3 but for 0, 8 and 16 T applied magnetic fields. As seen, the identical nature of the structure indicate zero-magnetic-field spin splitting. Solid line, 0 T; dotted line, 8 T; dashed line, 16 T.

However, a feature at 0.25 also appears as electrons only travel in one direction—left or right (L,R)—down the channel. This is robust as *V*_sd_ increases (Chen *et al.* 2008). This feature, which can be either plateau or resonance-like, is difficult to explain on any one-electron picture, as it indicates that the spin degeneracy has been spontaneously lifted. Confirmation of this is obtained by application of a strong magnetic field that is sufficient to separate the spins, but does not alter the shape or location of the 0.25 feature. This result is significant, as it shows that the feature is not the result of a reduction in transmission probability, but rather it implies a relationship between spin and momentum. It is noteworthy that, as the channel is widened, or carrier concentration increased, there is an absence of a feature at 0.5, showing that there is no other spin level associated with that direction of momentum. Chen *et al.* (2008) have developed a technique in which, by measuring the differential conductance, the energy levels can be completely followed as a function of both source–drain voltage and split-gate voltage. Denoting the direction of travel of the electrons contributing to the 0.25 as *R*_+_, i.e. carriers moving to the right, and their spin direction as +, then we would expect that a 0.5 would eventually be formed by *R*_−_, i.e. carriers also moving from the right but with the other spin direction; the energy difference between the + and − states is the spin gap (figure 9).

However, this does not occur no matter how far *V*_sd_ is increased, but when the L electrons enter the channel, so immediately do the R electrons, and the net result is a jump from 0.25 to 0.75, omitting the intermediate 0.5. We note that this has been observed for one-dimensional channels in InGaAs, but with GaAs, the 0.75 feature is located at 0.85, implying an extra offset that may be due to the exchange energy that varies with the carrier concentration. This result has implications for the emerging science of spintronics, where one of the goals is to control spin in the absence of a magnetic field. Although the phenomena in one-dimensional systems is only observed at low temperatures, a full understanding of the physics may lead to new systems being developed that show these effects at room temperature. Ihnatsenka & Zozoulenko (2009) have recently proposed a model based on spin-independent self-consistent Hartree calculations. It is argued that the 0.25 feature is due to nonlinear screening and pinning. It is unclear how this model could reproduce the observed magnetic-field dependence. On the other hand, we have found, by solving the Hartree–Fock equations self-consistently for a biased infinite parabolic wire with short-range *δ*-interactions (Lassl *et al.* 2007), that the system indeed magnetizes spontaneously already at zero magnetic field as subband fillings set in, i.e. very much like the unbiased case (Wang & Berggren 1996), but with 0.25 instead of 0.5 for the conductance.

Spin effects have been comprehensively studied in the context of the 0.7 structure, in particular, the tendency towards a spontaneous spin splitting with the minority spin pinned near the injecting (source) contact. As the separation of the one-dimensional subbands varies inversely with channel width, provided that the disorder is sufficiently small, it is possible to reduce the energy difference of the subbands to a value less than the Zeeman splitting of the spin levels in a moderate magnetic field of the order of a few Tesla. Under these circumstances, as the field increases, so the subbands split into two separate spin levels and the highest spin level of a subband is raised above the lowest spin level of the next most energetic subband. The characteristic of this overtaking has the form of a series of anti-crossings, which indicates repulsion between opposite spin levels.

**Figure 10.**
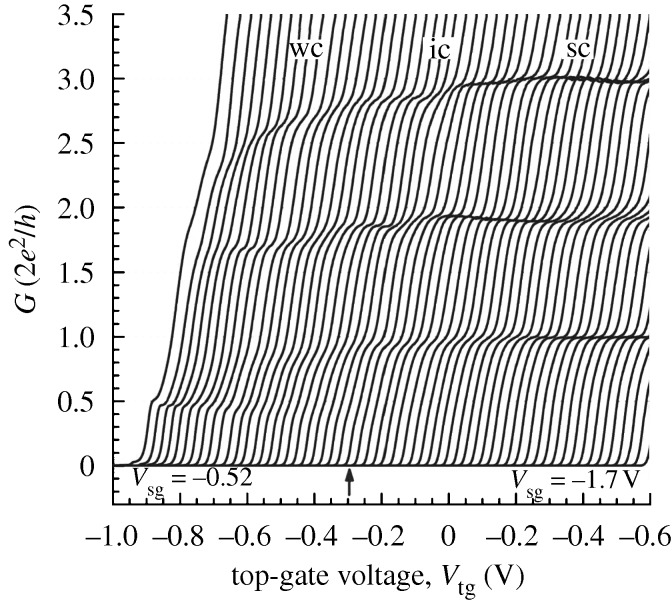
The device conductance at 80 mK as a function of a top-gate voltage that alters the carrier concentration, each plot is for a different split-gate voltage that alters the width of the channel. Decreasing carrier concentration is from right to left and three regimes of confinement are indicated sc, ic and wc for strong, intermediate and strong confinement, respectively. As the carrier concentration is decreased, so the 1 plateau disappears to be replaced by one at the 0.5 that is indicative of spin incoherence, the arrow marks an approximate start of the incoherent regime. Measurements on temperature dependence showed that lowering the temperature caused the return of spin coherence and the re-establishment of the 1 plateau.

For a considerable time, there have been theoretical investigations of the electronic properties of one-dimensional channels. As a consequence of the strong interaction between the carriers, a spin-charge separation occurs in which the spin and charge travel at different velocities. At low values of carrier concentration, the exchange energy between neighbouring electrons *J*, which varies exponentially with electron separation, is sufficiently small to become less than *kT*. Under these circumstances, the electrons, which although in a line do not approach closely due to the strong repulsion, have no preferred spin orientation, but rather the spin rotates randomly and is no longer a well-defined quantum parameter. This results in the conductance decreasing from 2*e*^2^/*h*, which is based on two distinct spin directions, to *e*^2^/*h* (Hew *et al.* 2008). However, as the carrier concentration increases, a plateau at 2*e*^2^/*h* is not necessarily observed. A spin wave that enters the channel is reflected back by a spin-incoherent ensemble (figure 10).

Applying a magnetic field such that the Zeeman splitting exceeds *kT* brings a reintroduction of the spin coherence and observation of conductance plateaux at *e*^2^/*h* and 2*e*^2^/*h*. Similarly, in the absence of a magnetic field, decreasing the temperature until *kT*<*J* will restore the spin coherence and plateaux at *e*^2^/*h* and 2*e*^2^/*h*.

**Figure 11.**
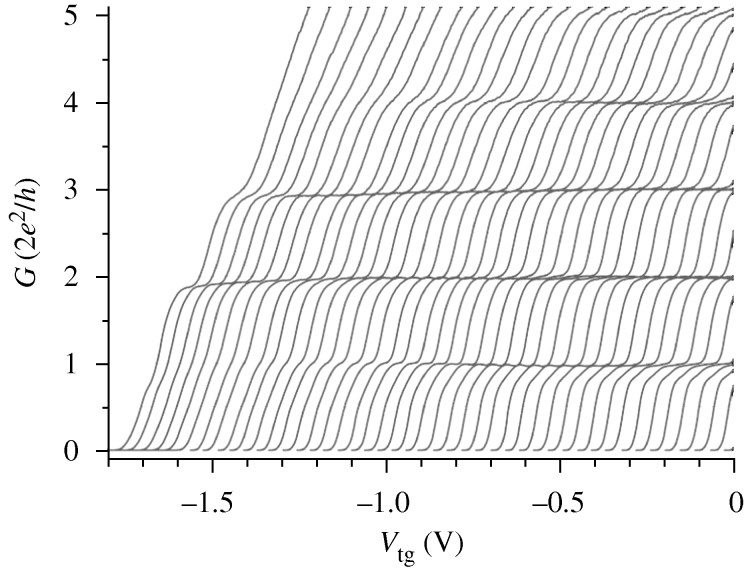
The device conductance is plotted against a top-gate voltage that lowers the carrier concentration from right to left. Each plot is for a different value of split-gate voltage that progressively widens the channel from right to left, and the overall carrier concentration is too high to enter the spin-incoherent regime at these temperatures. The ground state plateau at 1 disappears as the channel widens and the first plateau is then the 2, indicating a two-row spin-degenerate ground state.

A topic of current interest is the possibility of a Wigner lattice in the channel. When electrons form a single line or row, they are held in position by the confining electric field emanating from the split gates. Matveev and co-workers have investigated the resulting electron relaxation as the confining field is reduced (Meyer *et al.* 2007). In order to minimize the electron–electron repulsion, neighbouring electrons move sideways, thus increasing their mutual separation. This results in the formation of a zig-zag array, which when sufficiently pronounced, results in electrons that were previously second nearest-neighbours now becoming nearest-neighbours. This unusual situation can produce a number of possible spin phases; however, it is not clear how they can be experimentally observed, apart from the ferromagnetic phase. As the confining potential is further decreased, or the electron–electron repulsion increased, so the zig-zag array splits into two discrete rows that can hybridize if pushed together or be completely independent. The formation of two discrete rows has been observed by a jump in the conductance to 4*e*^2^/*h*, a result that cannot be explained on a subband picture where 2*e*^2^/*h* must always be the initial observation (figure 11; Hew *et al.* 2009; Smith *et al.* 2009).

A variety of spin effects have been observed in this strongly interacting regime, although, as yet, a ground state of three or more rows has not been found. The study of this aspect of the system in which relaxation in the second dimension is introduced is in its infancy, and flexible device structures are being developed to investigate it further.

## 5. Conclusions

Although a very simple device, the absence of disorder results in such a controllable channel being an ideal laboratory of quantum physics. Unanticipated spin effects have been observed, and techniques have been developed for following the energy levels as they move under the influence of a change in confinement potential and a magnetic field. In this review, we have concentrated on presenting the basic transport phenomena; thermal measurements, although difficult, promise to be of importance in future. We can expect more complex devices and measurement techniques to be developed, particularly devices that allow application of scanning-probe techniques to give greater understanding of the microscopic processes within the channel, especially in the weak confinement regime where the electron–electron interaction is of central importance. Here, a direct mapping of the distribution of the electrons is a realistic possibility. The nature of the spin–spin interactions is dependent on the absence of disorder and as other semiconductor technologies improve with time, we may expect that many-body physics will emerge there as well. Despite the large number of investigations, this subject is still in its infancy and as the technology of growth, processing and measurement improves, so much new physics may well be found as a consequence.

## Appendix A. Basics of conductance quantization and the AC and DC cases

In the case of a finite bias *V*_sd_ between source and drain, there is an important difference between AC and DC conductances, which may be used for measuring different features of the electron gas within the QPC. For simplicity, assume that the QPC is much extended, i.e. *ω*_*y*_≫*ω*_*x*_ in equation (2.2). For the moment, we may therefore assume that we deal with a very long straight quantum wire. There are different transverse subband states *φ*_*n*_(*y*), as well as freely propagating plane-wave states along the wire. The electrons thus occupy one-electron states of type

A1

Consider first the strictly one-dimensional case and assume *E*-normalization

A2

with energy . The velocity is  and the corresponding probability current density equals ±1/*h*, which also holds for one-dimensional *E*-normalized continuum states in general (Landau & Lifshitz 1959).

If there is a symmetric voltage drop *V*_sd_ between the two ends of the wire, there will be a net flow from source to drain (Martín-Moreno *et al.* 1992). The current *I*(*V*) set up in this way then derives from states within the energy window (*E*_F_−*eV*/2,*E*_F_+*eV*/2)=(*μ*_s_,*μ*_d_) in terms of the shifted chemical potential *μ*=*E*_F_. Assuming zero temperature and perfect transmission *T*(*E*)=1 for *E*>0, we thus obtain the current per spin direction

A3

when *μ*_d_>0. On the other hand, at sufficiently high bias *μ*_d_<0, we have

A4

because all states are unidirectional in this case.

The AC and DC conductances are obtained as *G*_AC_=∂*I*(*V*_sd_)/∂*V*_sd_ and *G*_DC_=*I*(*V*_sd_)/*V*_sd_. For *μ*_d_>0, the two conductances are evidently equal according to equations (A3) and (A4), i.e. *G*_AC_=*G*_DC_=*I*/*V*_sd_=*e*^2^/*h* per spin. In the common units *G*_0_=(2*e*^2^/*h*), we may write  per spin and 1 for the two spin directions.

In the nonlinear regime, the second case with *μ*_s_<0, there are profound differences. For the first *G*_*AC*_=*e*^2^/2*h* per spin, i.e.  in units of *G*_0_. This time, the DC conductance differs from the AC case as

A5

Here, Δ=*E*_F_+*eV*_sd_/2=*μ*_s_, which is directly related to the electron density in the wire. In summary, the two conductances *G*_AC_ and *G*_DC_ may be used for supplementary information about the wire: *G*_AC_ is related to quantized conduction and in what way a subband is occupied, while *G*_DC_ may be used to find the corresponding electron density via Δ*E*.

To generalize the results for the ideal one-dimensional case to a real wire, we include the transverse motions with subband energies ; the spin index *σ* is included since subbands may be Zeeman split or because of interaction-induced spin polarization. Hence, the subband dispersions equal . Assuming, as above, zero temperature and ideal transmission, we have, for a multi-subband wire,

A6

where the step function *Θ*(*x*)=1 for *x*>0 and zero otherwise. figure 5 illustrates the principal behaviour of the AC conductance of parabolic wire different band-fillings and source–drain bias for the case of spin-degenerate electrons. The DC conductance is obtained in the same way as

A7

where .

The above two expressions refer to ideal wires with step-like transmission cofficients . As mentioned, this is a good approximation for more extended QPCs with *ω*_*y*_≫*ω*_*x*_. However, the above picture also gives a good understanding for shorter QPCs. The main difference is that conductance features become less sharp when  is more gradual than the step function, for example, as for a hyperbolic QPC (Büttiker 1990).

Finite temperture *T* has similar effects. More generally, we therefore have for the current

A8

where  is the Fermi–Dirac function. The expressions for the AC and DC conductances may now be worked according to their definitions above. However, simplifications are usually introduced at this step. For example, variations of  and  with *V*_sd_ are ignored many times, i.e. basically one dismisses the variation of the QPC potential with *V*_sd_. Also, explicit effects of temperature, interactions (Lunde *et al.* 2009 and references therein), etc. on the QPC potential itself are usually simplified or omitted. Much theoretical work remains to be done here.

## Appendix B. Theoretical modelling of interaction-induced spin polarization and the question of bound states

As mentioned in the main text, a number of theoretical papers have been published, suggesting that a spin polarization does occur in QPCs and quantum wires, despite the theorem of Lieb and Mattis that states that the ground state of a one-dimensional system is non-magnetic. The intuitive argument in favour of spontaneous spin polarization is simple. At low conductances below the lowest plateau, the corresponding electron density in a QPC gets very low and eventually approaches zero with decreasing *V*_g_. This would favour parallel alignment of the spins because the electrons would gain exchange energy in this way. On the other hand, the kinetic energy increases with the alignment of spins. However, on lowering the density, the gain in exchange energy eventually wins and therefore induces spin polarization, even in the absence of a magnetic field. The spin polarization (magnetization) would be local, and it appears wherever the density is low. Based on these ideas, a first spin-polarized modelling based on the self-consistent Kohn–Sham LSDA (e.g. Parr & Yang 1989) was performed for an infinite parabolic wire right after the discovery of the 0.7 anomaly (Wang & Berggren 1996). Spontaneous spin polarization was found to take place every time a subband started to be occupied when increasing the Fermi energy. Strong variation of the effective *g*-factor was also found in qualitative agreement with more recent observations referred to above. The model has later been cast into a phenomenological split-band model for the 0.7 feature (Reilly 2005). The Reilly model accounts well for the conductance in long wires, shot noise signatures, etc. When extended to finite bias, we have found from the self-consistent Hartree–Fock solutions for *δ*-interactions that L and R states split separately in an infinite parabolic wire. Thus, spin polarization occurs when L and R subbands become occupied at *E*_F_±*V*_sd_. For zero temperature, this model predicts that the conductance would be 0.25 at high bias. On lowering the *V*_sd_, the conductance steps to 0.5, 0.75 and eventually to 1.0. For small values of *V*_sd_, the split L and R subbands overlap partially, which should result in more complex magnetization and conductance patterns. Such features need further studies.

The modelling for the infinite wire included only a local exchange potential *V*_ex_(*x*,*y*) in addition to *V*_conf_(*x*) in equation (2.1). It is well known that exchange-only LSDA models exaggerate spin-polarization effects. When Coulomb interactions are also included in the Kohn–Sham LSDA, the effect is to reduce the magnetization, but not to remove it. Exchange and correlation potentials *V*_ex_(*x*,*y*)+*V*_corr_(*x*,*y*) have therefore been fully incorporated in equation (2.1) for a series of subsequent models for infinite wires, as well as for QPCs with realistic geometries (Starikov *et al.* 2003; Jaksch *et al.* 2006; Lassl *et al.* 2007; Pepper & Bird 2008; Yakimenko & Berggren 2009; and references therein). The general picture that emerges from this type of model is that spontaneous spin polarization emerges in the relevant conductance regimes, i.e. also in the second subband (Starikov *et al.* 2003). For short wires, there is a structure in the vicinity of 0.7. For longer wires, the feature is shifted towards 0.5, which implies that a long wire is fully polarized at low densities (Jaksch *et al.* 2006), which may be of potential interest for spin filtering, etc. For long wires, one notices structures in the conductance that, at first sight, may remind us of resonances associated with, for example, bound states. However, the reason for such peak-like structures is that, as the polarization sets in, a rapid change in mobilities for the two spin directions shows up in the total conductance. Another important result is a pinning effect (Lassl *et al.* 2007). The persistence of a spin gap as an applied magnetic field is turned off also supports the idea of a spin gap (Koop *et al.* 2007; Yakimenko & Berggren 2009). In summary, all the LSDA models predict spontaneous spin polarization in relevant conduction regimes. However, we recall that LSDA models are strictly valid only at zero temperatures. In its present form, it will not be able to predict the activated behaviour observed in experiments, although there are cases for longer wires for which LSDA does predict the correct temperature dependence of the conductance (Jaksch *et al.* 2006; Sfigakis *et al.* 2008). Here, we may recall the virtual elevated state found by Starikov *et al.* (2003), but not further explored.

The presence of (quasi-) bound states in QPCs is, as we have seen above, an important issue in the Kondo scenario. Using the self-consistent LSDA, bound states have indeed been predicted (Hirose *et al.* 2003; Sushkov 2003; Meir 2008). However, for the realistic QPC structures referred to above, no (quasi-) bound states were obtained. On the other hand, there are charge and spin accumulations at the two ends of a QPC close to pinch off (Berggren & Yakimenko 2008). At the end, such features and bound states may be related to device geometry, as suggested by Sushkov (2003) and others. Recent quantum Monte Carlo calculations (Güçlü *et al.* 2009) of electron localization in a ring-shaped parabolic quasi-one-dimensional wire with a barrier also points to geometric effects. For a rectangular barrier, there is localization in the barrier region at pinch-off. The localization disappears, however, as the barrier is softened (cf. Yoon *et al.* 2009). Obviously more work is needed in the few-electron limit. At any rate, there seems to be little doubt that spin interactions are important for understanding the 0.7 conduction anomaly. Although LSDA easily yields spin polarization, it does not provide a full explanation, in particular, for the temperature-activated conductance. In the absence of an external magnetic field, the LSDA spin-polarized state is obviously highly degenerate with arbitrary directions for the interaction-induced magnetization. In such a situation, there should be spin-wave modes (Berggren & Yakimenko 2008) that become thermally excited with temperature. Like scattering from phonons (Seelig & Matveev 2003), such spin waves should lead to a reduction of the conductance with increasing temperature. The possibility of spin scattering has also been discussed by Tokura & Khaetskii (2002). The idea of spin-wave scattering is so far quite unexplored.

An important question is if the spin polarization within a QPC is static, as obtained from LSDA, or dynamic as suggested by the Kondo-like mechanism. After all, the LSDA is an approximation in which one writes the total wave function as a single Slater determinant. Spontaneous spin polarization means symmetry breaking, which, in turns, means that the simple approximation with a single determinant wave function breaks down. It is known, however, that a symmetry-broken LSDA state indictates what kind of correlations are inherent in the true state (Reimann & Manninen 2002). In our case, they are obviously of ferromagnetic type. Except for the Kondo-like, there are few attempts to go beyond the LSDA for our type of open systems. The reason is the complexity of the problem. However, there is a recent quantum Monte Carlo study of an infinite wire (Aryanpour & Han 2009). Linear conductance calculations suggest that the 0.7(2*e*^2^/*h*) anomaly results from a strong interaction of low-density conduction electrons to ferromagnetic fluctuations formed across the potential barrier. Although one cannot conclude from the calculations that there is a static ferromagnetic ground state, it is likely that it does not happen. However, it does not preclude an experimental observation that suggests a static magnetism, since what matters is the ratio of electron traversal time and the characteristic time of magnetic fluctuation. So without a true static magnetic ordering, it may still ‘look like’ one. If so, the two views, static versus dynamic polarization, are reconciled.
